# Bacteria-mammalian cell co-culture systems mediated by spatial engineering strategies

**DOI:** 10.3389/fbioe.2026.1999112

**Published:** 2026-10-21

**Authors:** Anqi Luo, Danshan Zhao

**Affiliations:** 1 School of Pharmacy, China Pharmaceutical University, Nanjing, China; 2 School of Food Science and Pharmaceutical Engineering, Nanjing Normal University, Nanjing, China

**Keywords:** bacteria, co-culture, hydrogels, microcapsules, spatial engineering

## Abstract

Bacteria and mammalian cells coexist and interact in many physiological and pathological environments, yet their direct *in vitro* co-culture is difficult because of mismatched growth rates, nutritional demands, and oxygen requirements. This Perspective discusses spatial engineering strategies that improve co-culture stability while preserving biologically relevant communication. We compare tumor spheroid colonization models, biomimetic hydrogel barriers, bacteria-containing microcapsules, layered hydrogel beads, and multiphasic droplets that support direct or indirect interactions. We further examine how material composition, crosslinking, pore size, shell thickness, mechanical stability, and compartment dimensions regulate bacterial confinement and molecular transport. Current systems still face trade-offs among leakage prevention, efficient exchange, physiological relevance, experimental duration, and throughput. Such models may enable mechanistic studies, therapeutic evaluation, and high-throughput investigation of dynamic bacteria-mammalian cell interactions under controlled conditions.

## Introduction

From human barrier tissues such as the intestine, respiratory tract, oral cavity, and skin to chronic infection sites and tumor microenvironments, mammalian cells and microorganisms are not two isolated types of living units. Instead, they co-exist over long periods and continuously interact within shared physicochemical spaces. Microbial colonization and microbial secretions can alter the proliferation, metabolism, immune responses, and drug sensitivity of animal cells, while the genetic background and physiological state of host cells can, in turn, shape the surviving microbial species and their functions. A recent study provided a striking example of this bidirectional interaction. Oncogenic KRAS mutations in colorectal cancer cells selectively enriched *Bacteroides acidifaciens*, while the bacterial surface protein SusF further amplified KRAS-MAPK signaling and promoted tumor growth by stabilizing the host protein ARHGEF2. This created a positive feedback loop in which host genetic alterations and microbial selection reinforced each other (Jiao et al., 2026). Similar interactions are also widely present in microbial colonization, tissue barrier damage, infection and drug resistance, biofilm formation, bacteria-mediated tumor therapy, and engineered bacteria-mediated cell sensing and drug delivery (Huang et al., 2022; Harimoto et al., 2022; Moya-Ramírez et al., 2022). To reveal such interdependent processes, the use of bacterial lysates, inactivated bacteria, cell-free culture supernatants, or separate monoculture systems is often insufficient. These approaches cannot reproduce the dynamic effects generated by continuous feedback between the two populations and therefore cannot fully reconstruct the underlying mechanisms. Thus, *in vitro* models that allow microorganisms and mammalian cells to coexist in the same controlled environment are needed, together with precise regulation of their spatial relationships and material exchange. However, placing live bacteria and mammalian cells directly in the same culture system often creates an asymmetric ecosystem that is difficult to maintain. Bacteria generally proliferate much faster than animal cells. They rapidly consume nutrients, alter pH and oxygen levels, and accumulate metabolic waste, toxins, or inflammatory stimuli. Mismatches in medium composition, oxygen requirements, culture duration, and spatial scale allow unconstrained direct co-cultures to become dominated by bacterial overgrowth, ultimately causing mammalian cell death and shortening the experimental window. Existing methods commonly improve the stability of animal cell-microorganism co-culture systems through Transwell inserts, antibiotic treatment, or continuous perfusion, but these strategies also introduce new limitations. Complete physical separation eliminates adhesion, invasion, and other contact-dependent processes. Antibiotics may alter bacterial states and directly affect mammalian cells. Continuous perfusion systems in microfluidic devices are increasing throughput and becoming a great asset where biomaterials can also be incorporated to create more relevant environments for the study of bacteria together with mammalian cells. However, they still require specialized equipment and fluidic control, and continuous flow may introduce shear stress or remove bacteria and soluble signals.

Hydrogel microspheres, layered microcapsules, and multiphasic microdroplets developed in recent years provide new spatial engineering strategies for animal cell-bacteria co-culture. Multilayer alginate or polyelectrolyte shells can confine bacteria within independent microenvironments while allowing the exchange of nutrients and metabolites (Moya-Ramírez et al., 2022; Jeong and Irudayaraj, 2023). Engineered hydrogels can reconstruct selective barriers between host cells and bacteria (Huang et al., 2022). Furthermore, the integration of polymer phase separation and photo-crosslinking allows liquid and hydrogel compartments to be combined within the same system. By changing the positions of cells in the core, shell, and interface, direct and indirect co-culture modes can be switched within a single platform (Alshareedah and Kumar, 2026). Although the host-associated microbiome includes bacteria, archaea, fungi, viruses, and other microorganisms, this Perspective focuses on the different spatial organizations of bacteria-mammalian cell co-culture models and compares their design logic and characteristics. An important limitation of this scope is that many host-associated bacteria remain difficult to isolate or maintain *in vitro* because of strict anaerobic requirements, specialized nutritional requirements, or dependence on other community members. Consequently, current co-culture models represent only the cultivable subset of the natural microbiota. These models include bacteria-encapsulating microspheres, core-shell or multilayer hydrogel structures, liquid-hydrogel interface systems, and tumor spheroid colonization models that do not depend on artificial shells, as schemed in Figure 1. We further discuss how hydrogel materials, crosslinking methods, shell thickness, pore and molecular retention properties, mechanical stability, and microsphere size jointly determine bacterial leakage, nutrient transport, metabolite exchange, physical contact, and culture duration. We believe that these co-culture platforms should not simply be understood as encapsulation tools for restricting bacterial growth. Instead, they should be regarded as spatial engineering strategies for regulating bacteria-cell interactions. Their real value lies in converting uncontrollable mixed co-cultures into adjustable microbial ecosystems. Nevertheless, existing models still have difficulty simultaneously ensuring stable long-term bacterial confinement, functional maintenance of both cell populations, and applicability to different combinations. Some systems also remain limited by antibiotic dependence and simple cellular compositions. Future work should improve model stability and biological complexity while maintaining experimental throughput, allowing the incorporation of more bacterial and host cell types and supporting continuous analysis of interaction processes and therapeutic responses.

**FIGURE 1:**
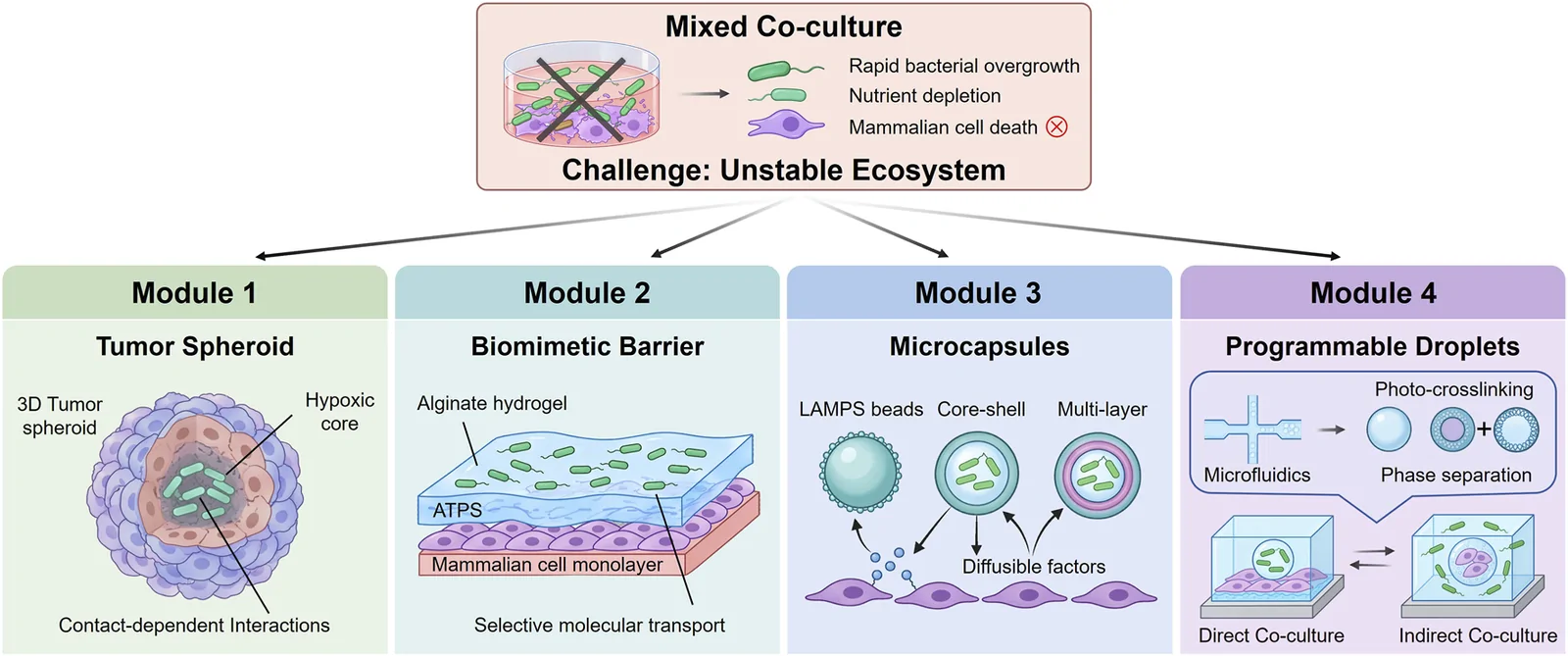
Spatial engineering strategies for stabilizing bacteria-mammalian cell co-culture through tumor spheroids, biomimetic barriers, microcapsules, and programmable droplets.

## Spatial organization strategies for bacteria-mammalian cell co-culture

The key differences among existing co-culture models lie not only in the materials or fabrication methods used, but also in where bacteria are positioned relative to host cells and which communication routes are retained between them. From autonomous colonization within tumor spheroids to complete hydrogel-mediated separation, these models provide different degrees of spatial constraint. However, the physiological relevance of a spatial arrangement depends on the target tissue and the research question. In models of intact mucosal barriers, a mucus layer or an engineered barrier that separates bacteria from epithelial cells is itself part of the normal tissue architecture. By contrast, studies of bacterial adhesion, invasion, biofilm formation, or intratumoral colonization require direct contact or tissue penetration. Stronger spatial constraints can improve culture stability but may exclude contact-dependent interactions, whereas weaker constraints retain these interactions but increase the risk of bacterial overgrowth. Spatial organization should therefore be selected to reproduce the contact mode of the target biological process while providing sufficient experimental control. Table 1 lists representative bacteria and mammalian cell co-culture systems and compares their cell types, materials, fabrication methods, spatial relationships, and applications.

**TABLE 1:** Comparison of representative bacteria-mammalian cell Co-culture systems.

Bacterial species	Cell type	Materials	Fabrication method	Spatial relationship	Co-culture duration	Application	References
*Escherichia coli* *E. coli* Nissle 1917 (EcN)	A549	PEG-dextran	Microfluidic droplet generation combined with liquid-liquid phase separation (LLPS) and UV photo-crosslinking	Direct and indirect co-culture	27 h	High-throughput screening of host-microbe interactions	Alshareedah and Kumar, 2026
*Salmonella typhimurium*, *Listeria monocytogenes*, *Proteus mirabilis*, *E*. *coli*	CT26, 4T1, MC26, HT29	Three-dimensional tumor spheroids	Direct inoculation of bacteria into tumor spheroids	Bacteria colonize the hypoxic and necrotic core of the tumor	2 weeks	Microbial anticancer therapy	Harimoto et al., 2022
*P. aeruginosa*, *Shigella flexneri*	16-HBE, Caco-2	ALG-MUC and PEG-DEX aqueous two-phase system (ATPS)	Formation of a mucus-like gel on the cell monolayer through calcium chloride crosslinking	Hydrogel barrier separates the cell monolayer from bacteria	12 h	*In vitro* pathological models of respiratory and intestinal mucosal barriers	Huang et al., 2022
*L*. *lactis*	HCT116, HepG2, A498	Alginate core-shell hydrogel beads	Preparation of an alginate core by air extrusion, followed by outward gelation in calcium chloride to form a protective shell	Indirect co-culture	48 h	Evaluation of antitumor bioactivity *in vitro*	Jeong et al., 2024
*E*. *coli*, *B*. *subtilis*, *L*. *lactis*, *P*. *aeruginosa*	HCT116	Multilayer hierarchical alginate hydrogel beads	Needle extrusion combined with alternating interfacial gelation using low-viscosity alginate	Indirect co-culture supporting multilayer spatial distribution	12 h	Microbial quorum-sensing communication	Jeong and Irudayaraj, 2023
*E*. *coli*	Flp-in CHO, IgG-C CHO	A multilayer polymer shell consisting of an alginate core, poly-L-lysine, and an outer alginate layer	Crosslinking of the sodium alginate core, followed by alternate dip-coating in PLL and alginate, with the outer layer crosslinked using barium ions	Indirect co-culture	20 h	Real-time metabolite monitoring using living-cell biosensors	Moya-Ramírez et al., 2022
*P. aeruginosa*	A549	PDMS microwell-array chip	Fabrication of a multilayer microchip, followed by culture of 3D tumor spheroids and continuous perfusion with bacteria for infection	Bacterial biofilms colonize and cover the tumor spheroid surface	8 h	Investigation of iron metabolism and ferroptosis resistance in the tumor microenvironment	Yeung et al., 2024

Tumor spheroid colonization models impose the least artificial spatial restriction on bacteria. Harimoto et al. first brought bacteria into contact with tumor spheroids and subsequently removed bacteria outside the spheroids through washing and gentamicin treatment, allowing bacteria within the hypoxic and necrotic regions to continue growing. This model could be maintained for more than 2 weeks and was used to observe the process of bacterial colonization (Harimoto et al., 2022). Yeung et al. formed *Pseudomonas aeruginosa* biofilms on lung tumor spheroids cultured in a microfluidic system and found that bacterially secreted siderophores altered local iron metabolism and reduced the sensitivity of tumor cells to ferroptosis (Yeung et al., 2024). These models preserve bacterial adhesion, invasion, and biofilm formation and are therefore suitable for studying contact-dependent interactions. However, the final bacterial location is mainly determined by oxygen and nutrient gradients within the tumor spheroid rather than being predefined by the experimenter. Culture outcomes are also affected by bacterial strain characteristics, antibiotic sensitivity, and tumor cell type.

Introducing a biomimetic barrier between host cells and bacteria can improve culture stability without completely blocking molecular exchange. Huang et al. covered a mammalian cell monolayer with an alginate-mucin hydrogel and then used an aqueous two-phase system to confine bacteria to defined regions, thereby reconstructing a mucus-like interface (Huang et al., 2022). Notably, the hydrogel was not an inert isolation material in this system. The addition of mucin changed the diffusion properties of the gel and increased bacterial tolerance to antibiotics. This finding indicates that the artificial microenvironment itself may affect bacterial phenotypes. Material composition therefore determines not only whether the model can operate stably but also the biological effects ultimately observed. The bacteria-mammalian cell interface may comprise host-derived mucus or extracellular matrix, bacteria-derived extracellular polymeric substances (EPS), and an engineered hydrogel. EPS production and composition vary with bacterial strain and culture conditions and, when present, can affect bacterial adhesion and retention, molecular diffusion, antibiotic penetration, and host-cell exposure to bacterial products. Because bacterial EPS is dynamically produced and regulated, it should not be considered equivalent to an externally supplied hydrogel, even when the two coexist at the same interface (Liu et al., 2022). Another approach is to confine bacteria within independent microspheres while mammalian cells remain in the external culture environment. Moya-Ramírez et al. encapsulated an *Escherichia coli* biosensor within an alginate core and reduced bacterial escape through polyelectrolyte coating. The bacteria could detect lactate produced by external CHO cells without disrupting the mammalian cell culture through rapid proliferation (Moya-Ramírez et al., 2022). Jeong and Irudayaraj further constructed layered hydrogel microspheres with adjustable shell thicknesses, allowing different bacteria to be positioned in the core or peripheral layers and communicate through diffusible signals (Jeong and Irudayaraj, 2023). In a subsequent study, *Lactococcus lactis* expressing β-glucuronidase was encapsulated within a hydrogel shell. The enzyme produced by the bacteria could diffuse outward and activate an antitumor prodrug, which then acted on two-dimensional cancer cells or three-dimensional tumor spheroids (Jeong et al., 2024). A common advantage of these models is that they decouple bacterial proliferation from host cell culture while retaining the exchange of metabolites and enzymes. However, they are more suitable for studying effects mediated by diffusible factors and cannot readily reproduce processes requiring bacterial adhesion to or invasion of host cells. Multiphasic droplets provide a more flexible connection between direct and indirect co-culture. Alshareedah used polymer phase separation to form liquid and hydrogel compartments. In the direct co-culture configuration, bacteria grew within the liquid core, while mammalian cells adhered to the hydrogel-liquid interface and could therefore contact the bacteria. Following sequential encapsulation, mammalian cells were embedded within the hydrogel, while bacteria were distributed in the peripheral liquid phase, allowing communication only through diffusion (Alshareedah and Kumar, 2026). Switching between these two configurations on the same platform allows direct assessment of whether a phenotype depends on physical contact. This comparative capability is more meaningful than simply extending culture duration because it converts spatial relationships into independently adjustable experimental variables.

Evaluation of these systems should extend beyond bacterial proliferation and molecular transport to mammalian cell responses, including viability and proliferation, epithelial barrier integrity, inflammatory signaling, metabolic state and iron homeostasis, apoptosis, and drug sensitivity. For example, the alginate-mucin barrier used by Huang et al. protected the underlying 16-HBE and Caco-2 monolayers, with mammalian cell viability influenced by the hydrogel formulation (Huang et al., 2022); *Pseudomonas aeruginosa* siderophores altered tumor-cell iron homeostasis and reduced ferroptosis sensitivity (Yeung et al., 2024); and Lactococcus lactis-mediated prodrug activation reduced cancer-cell viability and induced apoptotic signaling in monolayers and tumor spheroids (Jeong et al., 2024). A co-culture system should therefore be considered stable only when the intended functions of both the bacterial and mammalian cell populations are maintained. In summary, no co-culture structure is optimal independently of the research question. Direct contact should be retained when studying bacterial colonization or biofilms. Material confinement can provide a more stable observation window when the focus is on metabolites, enzymes, or engineered bacterial signaling pathways. If the mechanism of action is unclear, direct and indirect co-culture should be compared under the same material and culture conditions.

## Coordinated regulation of bacterial confinement and mass transfer

In spatially confined co-culture systems, bacterial retention and molecular transport must be considered together. If the barrier is too loose, bacteria can escape and dominate the culture system. If the barrier is too dense, the exchange of nutrients and signaling molecules becomes restricted. Alginate can be crosslinked under mild conditions and is suitable for encapsulating live bacteria, but a single-layer gel is often insufficient to prevent long-term bacterial escape. Moya-Ramírez et al. improved microsphere stability through chitosan or polylysine coating, with the polylysine layer providing stronger resistance to swelling. However, increasing the number of coating layers also delayed lactate transport. The sensing response of the double-layer structure occurred approximately 2 hours later than that of the single-layer structure (Moya-Ramírez et al., 2022). The layered alginate microspheres constructed by Jeong and Irudayaraj similarly showed that the outer shell could reduce leakage and support longer culture periods, although oxygen and nutrient gradients caused uneven bacterial growth (Jeong and Irudayaraj, 2023). Material barriers can also achieve selective transport by exploiting differences in molecular size. Jeong et al. confined β-glucuronidase-expressing *Lactococcus lactis* within alginate microspheres, allowing the precursor compound to enter the gel and be converted before the active product acted on external cancer cells (Jeong et al., 2024). This system retained not only bacteria but also enzymes with relatively large molecular sizes. However, the molecular retention properties of a gel cannot be determined solely by molecular weight. Molecular conformation, charge, and gel swelling also affect transport. Compared with ionically crosslinked natural hydrogels, PEG hydrogels allow network structure to be adjusted by changing polymer concentration, branching degree, and crosslinker length, making pore size, stiffness, and swelling behavior easier to design according to culture requirements. Alshareedah estimated a pore size of approximately 4–10 nm in a 10% PEG gel. A 10 kDa dextran could slowly enter the gel, whereas the transport of 70 kDa dextran and bovine serum albumin was clearly restricted. The authors further compared the effects of different PEG concentrations on A549 cell growth. A 5% gel supported spheroid formation, whereas a 10% gel failed to support spheroid growth because the denser network imposed stronger physical constraints. In the indirect co-culture configuration requiring complete encapsulation of host cells, the authors increased PEG branching and shortened the crosslinker to obtain a stiffer gel, reduce swelling after hydration, and maintain spatial separation (Alshareedah and Kumar, 2026).

Changes in material composition do not necessarily produce corresponding changes in molecular diffusion. Huang et al. found that adding mucin altered the viscoelasticity of alginate gels and improved the stability of the aqueous interface, but the effective diffusion coefficients of 150 kDa dextran and IgG did not differ significantly among the gels (Huang et al., 2022). This indicates that material rheology and interface stability cannot be used directly to infer transport capacity. When evaluating barrier performance, the diffusion behavior of the metabolites, proteins, or drugs that actually participate in the interaction must be measured separately.

Structural size mainly affects material exchange by changing diffusion distance, available culture volume, and the surface-area-to-volume ratio. Alshareedah found that mammalian cell viability after 48 h was higher in 90 μm droplets than in droplets of approximately 50 μm. This suggests that increasing droplet volume can slow nutrient depletion and metabolite accumulation, although it reduces compatibility with conventional flow cytometric sorting (Alshareedah and Kumar, 2026). In tumor spheroid models, once the diameter reaches approximately 500 μm, oxygen diffusion into the interior becomes insufficient to compensate for cellular consumption, resulting in a hypoxic core that favors bacterial colonization (Harimoto et al., 2022). Structural size is therefore an important variable determining molecular transport distances and local concentration gradients. Biomaterial selection and device architecture can also be used to regulate oxygen transport. Gas-permeable materials, controlled barrier thickness and permeability, separately perfused oxic and anoxic compartments, and integrated oxygen sensors can generate and monitor oxygen gradients that support both oxygen-dependent mammalian cells and obligate anaerobes (Jalili-Firoozinezhad et al., 2019). The target oxygen profile should be selected according to the tissue context because mucosal, tumor, and infection models require different oxygen conditions.

## Perspective

Taken together, these studies show that the central challenge in bacteria-mammalian cell co-culture remains the use of spatial engineering strategies to balance confinement, effective material transport, and physiological activity. Strict confinement helps control bacterial overgrowth but may weaken normal material exchange. Improving physiological relevance generally requires more complex cellular compositions and microenvironments, while increased complexity reduces experimental controllability, reproducibility, and throughput. At the same time, most existing conclusions are based on a limited number of bacterial strains and host cell combinations. Different bacterial species, tissue sources, and culture conditions often require renewed optimization. The field therefore still lacks a general design framework that can coordinate long-term stability, quantitative analytical capability, and scalability of application. Future technological advances will focus on improving the designability of confinement and material transport. Hydrogels must resist swelling and bacterial degradation while retaining defined transport pathways for target metabolites, proteins, or drugs. Material transport parameters should also be established using actual effector molecules rather than a single tracer. Medium composition, the oxygen transport capacity of the carrier liquid, and droplet size must be optimized together to extend co-culture duration. Spatial structures should shift from fixed configurations to modular designs so that direct contact, selective separation, and different transport distances can be compared under the same conditions. As these platforms become further integrated with high-throughput sorting, real-time imaging, and sequencing analysis, the study objects are expected to expand from single bacterial strains and cell lines to multispecies communities and tissue models containing immune cells, stromal cells, and patient-derived cells. Reducing dependence on antibiotics and establishing standardized methods for evaluating bacterial leakage, molecular transport, and host cell function will also be essential for improving model reproducibility and general applicability. When bacterial spatial position, contact mode, and material exchange can be controlled independently, co-culture models will no longer be only systems in which 2 cell populations are cultured together. They will become experimental systems for resolving causal relationships and dynamic feedback. These models can bridge simplified monoculture experiments and complex animal models, providing more interpretable research tools for infection mechanisms, tumor microbiology, engineered bacterial therapy, drug evaluation, and biomanufacturing.

## Data Availability

The original contributions presented in the study are included in the article/supplementary material, further inquiries can be directed to the corresponding author.
